# Genetic analysis and epidemiology of Crimean Congo hemorrhagic fever viruses in Baluchistan province of Pakistan

**DOI:** 10.1186/1471-2334-13-201

**Published:** 2013-05-04

**Authors:** Muhammad Masroor Alam, Adnan Khurshid, Salmaan Sharif, Shahzad Shaukat, Muhammad Suleman Rana, Mehar Angez, Syed Sohail Zahoor Zaidi

**Affiliations:** 1 Department of Virology, National Institute of Health, Chak Shahzad, Park Road, Islamabad, 44000, Pakistan

**Keywords:** Crimean Congo hemorrhagic fever, Pakistan, CCHFV Asia type-1 genotype, Baluchistan, Molecular epidemiology

## Abstract

**Background:**

Pakistan is considered as an endemic country for Crimean-Congo Hemorrhagic fever with numerous outbreaks and sporadic cases reported during the past two decades. Majority of cases are reported from Baluchistan province with subsequent transmissions to non-endemic regions mainly through infected animals directly or via infested ticks. We hereby describe the molecular investigations of CCHF cases reported during 2008 in Quetta city of Baluchistan province.

**Methods:**

Serum Samples from 44 patients, with clinical signs of hemorrhagic fever attending a tertiary care hospital in Quetta city, were collected and tested for CCHF virus antigen and genomic RNA, using capture IgM EIA kit and standard RT-PCR assay, respectively. The partial S-gene fragments were directly sequenced to get information related to the prevailing CCHFV genotypes and their molecular epidemiology in Pakistan.

**Results:**

Out of the total forty four, sixteen (36%) samples were found positive for CCHF IgM. Similarly, viral RNA was detected in six (16%) samples. Phylogenetic analysis revealed that all study viruses belong to genotype Asia-1 with closest similarity (99-100%) to the previously reported strains from Pakistan, Afghanistan and Iran.

**Conclusion:**

We conclude that CCHF virus remains endemic within Baluchistan and its neighboring regions of Afghanistan warranting a need of incessant surveillance activities.

## Background

Crimean Congo Haemorrhagic Fever (CCHF) virus belongs to genus *Nairovirus* within the family *Bunyaviridae* with a triple-segmented RNA genome. The Large or L segment encodes non-structural proteins which comprise the RNA dependent RNA polymerase, and the medium (M) and small (S) segments encode structural proteins for the surface glycoproteins and nucleocapsid respectively [1]. Although the disease was originally notified as febrile illness in the Western Crimea during 1940s, the etiological viral agent was not identified and reported until much later when a similar disease was reported from Congo in 1956, leading to its name as Crimean-Congo haemorrhagic fever in 1970s [2]. The family *Bunyaviridae* contains 5 genera including *Orthobunyavirus, Hantavirus, Phlebovirus, Nairovirus* and *Tospovirus* with more than 300 species [3]. The genus *Nairovirus* currently contains 35 distinct viruses of which only three are known to cause disease; CCHF, Dugbe and Nairobi sheep disease virus [4]. All members of the genus *Nairovirus* are believed to be transmitted mainly through hard ticks especially species of the *Hyalomma*, *Rhipicephalus* and *Dermacenter*[4]. CCHF virus (CCHFV) only causes disease in humans and the incubation period ranges from 2–9 days depending upon the route of exposure and viral inoculums [5]; 3.2 days after tick bite, 5 days after contact with infected livestock blood or tissue and 5.6 days after contact with infected human blood [6]. Several important factors are thought to contribute towards CCHF disease spread such as trade and exchange in livestock [4,7], transmission via migratory birds infested with infected ticks [8] and subsequent infection of new hosts [4,9]. When infected ticks are introduced to new ecological zones, differences in tick feeding preferences and vertebrate host availability also contribute to new transmission cycles [10]. Transmission in nosocomial settings also contributes to the spread of CCHFV [11].

**Figure 1:**
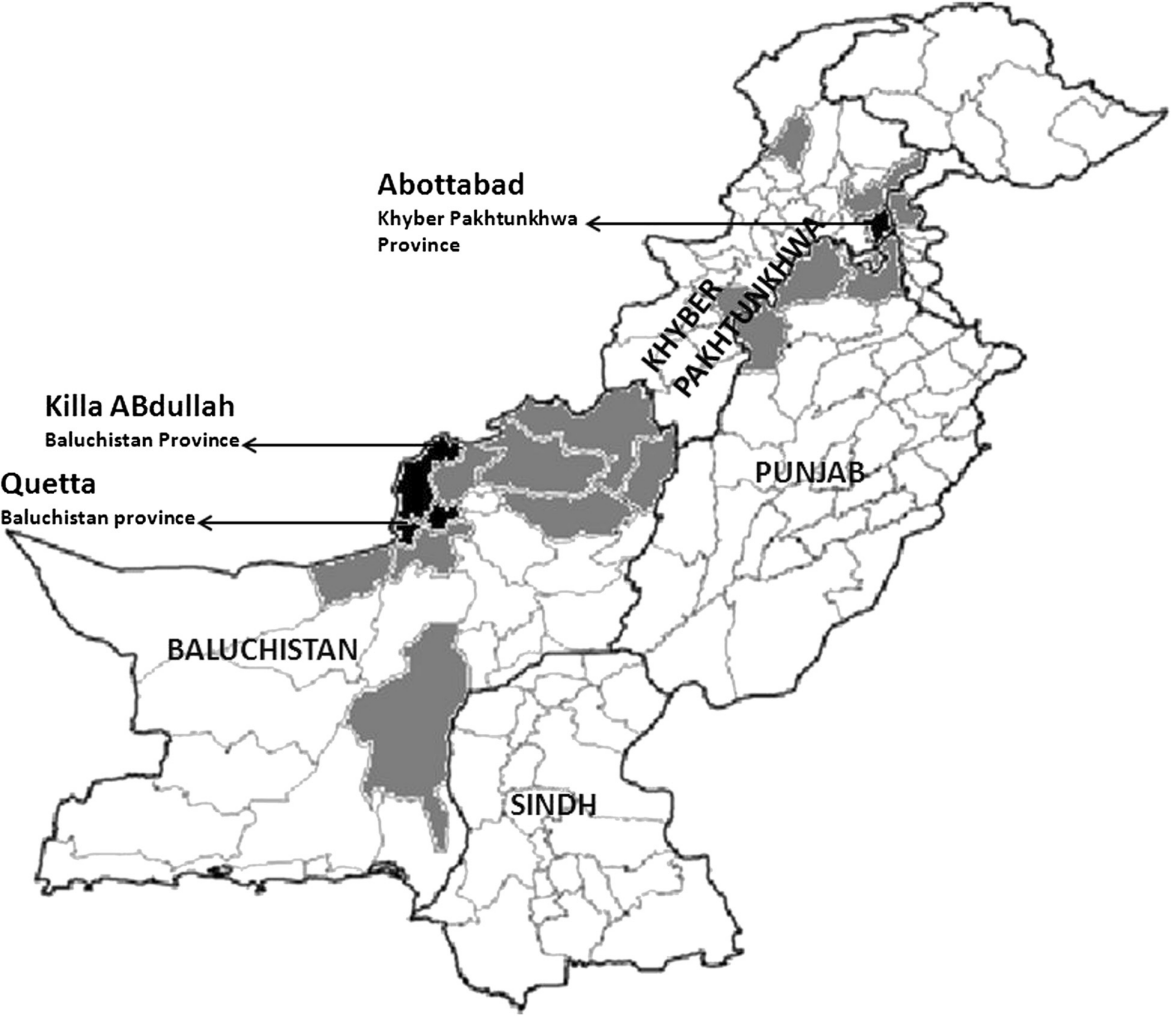
**Geographical map of Pakistan showing districts with IgM confirmed CCHF cases found during the years 2003 to 2008 reported to National Institute of Health, Islamabad, Pakistan.** Districts with proportion of case burden have been indicated through black (high disease burden districts) and grey color (low disease burden non-endemic districts with sporadic cases). Majority of IgM confirmed cases (n = 57) found during 6 years (2003–2008) belong to Baluchistan province, followed by Khyber Pakhtunkhwa (n = 20); Punjab (n = 6); Sindh (n = 02). The least number of cases from Sindh province indicates low prevalence of CCHF infection in this province as well as the fact that Karachi, the capital of Sindh province, also contains the diagnostic facilities so the suspected cases are not referred to National Institute of Health, Islamabad.

Previous reports have documented a wide geographical distribution of CCHF virus ranging from Eastern Asia to Africa and Europe with 15-60% mortality rates [3]. The first CCHF case in Pakistan was reported in 1976 from a patient who underwent laparotomy at Rawalpindi General Hospital (13). Since 1976 to 2003, 14 epidemics, including 8 with nosocomial origin, have been reported in the country, mainly in the Western and North Western areas [11] (Figure 1). In Baluchistan province, the first sporadic case was documented in 1978, whereas recorded outbreaks have occurred yearly since 1987 and then in 1994, 1995, 1998, 2000 and 2001 [12,13]. Thus it has been now known that CCHF virus circulates locally in Baluchistan [14] that has been declared as the disease endemic area in Pakistan [12]. Confirmed CCHF cases have also been reported from patients residing in Afghanistan as they usually seek treatments in hospitals of Quetta city, the capital of Baluchistan which borders Afghanistan.

The National Institute of Health, Islamabad serves as the country’s focal laboratory center receiving thousands of different diagnostic samples including those from CCHF suspected cases across the country. Until recently, the diagnosis of CCHF had been conducted through serological testing using the CCHF Capture IgM ELISA; however, very recently, the molecular diagnostic services have been initiated at the institute’s Department of Virology. We hereby describe the application of such improved diagnostic facilities for the molecular detection and genetic diversity of CCHF viruses detected in six patients admitted to Fatima Jinnah General and Chest (FJG&C) Hospital, Quetta.

## Methods

The concept and design of the study was approved through the Pakistan’s National Institute of Health Internal Review Board. This work was conducted on archived samples received during 2008 that were stored at −80°C. Previously, the results of these samples were reported on the basis of IgM detection and only positive samples were kept stored. We processed all such samples for the molecular detection of viral RNA and further characterization. A total of forty four serum samples from suspected patients were received from FJG&C hospital, Quetta during September to October 2008 and were available for this study with the demographic details of patients as given in Table 1. The patients were admitted to the hospital as they fulfilled the case definition criteria for CCHF. Standard case definitions were practiced as laid by the World Health Organization. A “suspected” case is defined as any patient with sudden onset of illness with high-grade fever over 38.5°C for more than 72 hours and less than 10 days, especially in CCHF endemic areas and among those in contact with sheep or other livestock. A “probable” case is defined when any patient with acute history of febrile illness for 10 days or less accompanied with thrombocytopenia less than 50,000/mm^3^ and presence of any two of the signs like petechial or purpuric rash, epistaxis, haematemesis, haemoptysis, blood in stools, ecchymosis, gum bleeding, or other hemorrhagic symptoms, however, diagnosis of a “confirmed” case is made if any probable case with positive diagnosis of CCHF markers in blood sample such as presence of IgG or IgM antibodies in serum and/or detection of viral nucleic acid in specimen. The formal consent was obtained from the subjects on a standard patient clinical records chart, however, the individual identity was kept anonymous while dispatched for the laboratory procedures.

**Table 1:** Demographic details of CCHF confirmed patients admitted at Fatima Jinnah General and Chest Hospital, Quetta

**S. No.**	**Country of origin**	**Signs and symptoms**	**Platelet count/ul of blood**	**IgM ELISA**	**RT-PCR**
1	Pakistan	Fever, Headache and Nose bleeding	30000	Positive	Negative
2	Pakistan	Fever and Nose bleeding	27000	Positive	Negative
3	Afghanistan	Fever and Nose bleeding	36000	Positive	Negative
4	Pakistan	Fever and Nose bleeding	70000	Positive	Positive
5	Afghanistan	Fever and Nose bleeding	23000	Positive	Negative
6	Pakistan	Fever, Headache and Nose bleeding	28000	Positive	Positive
7	Pakistan	Fever and Nose bleeding	31000	Positive	Negative
8	Pakistan	Fever and Nose bleeding	35000	Positive	Negative
9	Afghanistan	Fever and Nose bleeding	29000	Positive	Negative
10	Pakistan	Fever, Headache and Nose bleeding	60000	Positive	Positive
11	Afghanistan	Fever, Headache and Nose bleeding	73000	Positive	Negative
12	Pakistan	Fever, Headache and Nose bleeding	34000	Positive	Negative
13	Pakistan	Fever and Nose bleeding	16000	Positive	Positive
14	Pakistan	Fever and Nose bleeding	74000	Positive	Negative
15	Pakistan	Fever and Nose bleeding	63000	Positive	Positive
16	Pakistan	Fever and Nose bleeding	47000	Positive	Positive

Area of origin of patients where he/she encountered the disease has been given.

Unfortunately, the information of disease outcome was not recorded. RT-PCR = Reverse Transcriptase-PCR results based on S-gene primers used to detect CCHF virus RNA.

All of the sixteen IgM positive samples were processed for detection of viral RNA through RT-PCR. RNA was extracted using QIAamp viral RNA minikit (Qiagen GmbH, Germany) as per standard instructions. CCHFV RNA was detected by the specific amplification of a fragment of the viral S-segment using the protocol of Tino *et al.,* 1996 [15]. Briefly, One step RT-PCR reaction was performed with 1X PCR buffer, 0.4 pmol/ul of each primer (F2 and R3), 0.2 mM of each dNTPs, 21 units of AMV-reverse transcriptase enzyme, 20 units of RNase inhibitor with 5 units of *Taq* DNA polymerase. The reaction tubes containing 100ul reaction mix were subjected to 42°C for 45 minutes followed by 40 cycles of 94°C for 40 seconds, 38°C for 40 seconds and 72°C for 90 minutes. The second round was performed with the same reagent concentrations using the primers F3 and R2 but without reverse transcriptase and RNAase inhibitor enzymes and the annealing temperature was changed to 41°C. Amplified PCR products were then directly sequenced on ABI automated Genetic analyzer using Big dye terminator cycle sequencing reaction kit v3.1. CCHFV sequences obtained in this fashion were analyzed with Sequencher software (version 4.9; GeneCodes Corporation, USA) and contigs were constructed from consensus sequences. Multiple sequence alignments were performed with the ClustalW in MEGA 4.0 software. Alignments were generated with default parameters and adjusted manually. Distance matrices and phylogenetic analyses were conducted using MEGA 4.0. The dendograms were drawn by Neighbor Joining method using Kimura 2-parameter model for estimating nucleotide sequence distances. The partial S-gene sequences obtained in this study have been submitted to the GenBank under accession numbers KC869988 to KC869993.

## Results

The forty four serum samples received from FJG&C hospital Quetta were all tested for CCHF IgM. Sixteen samples (36%) were found positive for IgM (specific to CCHFV antigen) whereas viral RNA was detected in six (16%) samples. The mean age of IgM positive patients was 30 years (range; 12–65) including 12 males and 4 females. There was no significant difference found between age and gender in relation to CCHF IgM positive results (*P > 0.005*).

**Figure 2:**
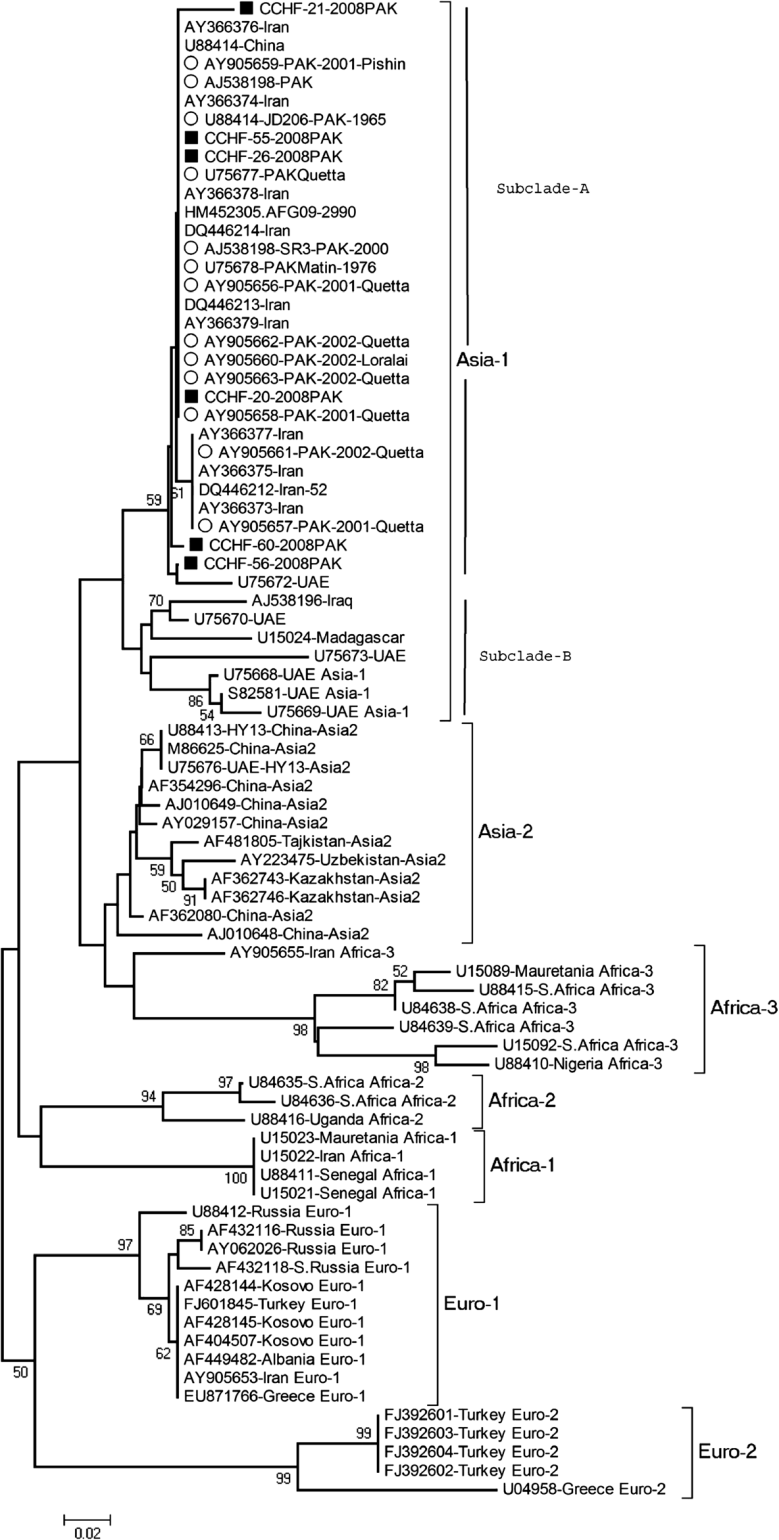
**Phylogenetic analysis and reconstruction of genetic tree based on partial S-gene segment generated through Kimura-2 parameter using the Neighbor Joining model.** Horizontal branch lengths represent number of nucleotide differences between taxa (individual virus sequence). Number at the nodes indicates bootstrap values shown above 50 to demonstrate the robustness of grouping using 1000 datasets replicas. Viruses from 7 genogroups (Asia-1, Asia-2, Euro-1, Euro-2, Africa-1, Africa-2 and Africa-3) are represented. Asia 1 genogroup constitutes viruses previously reported from Pakistan, Afghanistan, Iran, UAE, Madagascar and Iraq. The dark squares represent viruses from this study while open circles indicate viruses detected from Pakistan in previous years.

The partial S-gene segments amplified by RT-PCR were sequenced to determine the genetic diversity of CCHFV strains and their relationship with the already reported viruses from Pakistan and other regions. 220 bp obtained from the six sequences were found to belong to Asia-1 genotype with closest match (99-100%) to CCHF viruses reported previously from Pakistan, Afghanistan and Iran in contrast to Iraq and Madagascar Asia-type 1 viruses (Figure 2).

The phylogenetic reconstructions distinguished the Middle East Asian viruses into two distinct sub-clades within Asia-1 genotype. Subclade “A” represents viruses from Pakistan, Iran, Afghanistan and China while subclade “B” comprises viruses from Iraq, Madagascar and United Arab Emirates (UAE). All of the CCHFV strains isolated from Pakistan fall under Clade-A having 99-100% similarity with viruses from Afghanistan, Iran and China.

The pairwise nucleotide distances based on the number of substitutions per site were calculated using MEGA 4.0. The CCHF virus strains collected in Pakistan during 2008 had 98-100% similarity among each other and to strains reported from Afghanistan (HM452305), China (U88414) and Iran (AY366373-79). These viruses showed a higher level of divergence (5-16%) to the Asia-1 Clade-B viruses.

## Discussion

This study describes the epidemiological and genetic investigation of CCHF viruses detected in serum samples of clinically suspected and subsequently laboratory confirmed patients, received during September to October, 2008. The study subjects include patients from Pakistan and Afghanistan that were admitted to Fatima Jinnah General and Chest Hospital, Quetta.

In Pakistan, CCHFV is endemic to many areas of Baluchistan that harbor ticks and appropriate vertebrate hosts which remain in close contact with humans. Geographically, Baluchistan is the largest province of Pakistan and occupies area of 348,189 km^2^ with hyper-arid climate. Livestock farming is the main work practice and source of income for majority of the population. 93% of the provincial land area comprises rangelands that cannot sustain vegetation throughout the year, leading to seasonal migrations of nomadic populations to areas within the Baluchistan province and bordering areas of Afghanistan along with their livestock in search of food and water for themselves as well as their cattle and sheep flocks. These animals may serve as disease carrier or carry CCHFV infected ticks and become a major source of transmission across their migratory places.

Although the CCHF infection has been reported in Pakistan since 1976, the available data on CCHF cases during the past few years is very limited. It is most likely that many of cases have not been reported due to lack of proper surveillance system in Pakistan. This concern has been raised by previous studies [12,16] highlighting an unremitting need of surveillance, laboratory and medical facilities, especially in the CCHF endemic areas. This insufficiency in health services has led to alternative diagnostic measures based on clinical presentations and disease manifestations in patients. Although clinical diagnosis is useful, the high mortality of CCHF and propensity for nosocomial transmission warrant rapid laboratory confirmation to control and minimize epidemics.

*Hyalomma* tick species are considered as the main transmission vector in European as well as Asian and African countries especially *H. marginatum marginatum* and *H. analiticum analiticum*[8] however, there have been reports of other tick species found positive for CCHF viruses like *Ornithodoros lahorensis*, *Rhipicephalus sanguineus*, *Rhipicephalus bursa*, *and dermacenter* species [17]. For instance, CCHF virus was isolated from *Boophilus* ticks in Madagascar, while a Greek isolate of the virus was recovered from *Rhipicephalus*[18]. In admonition to the role of ticks, there have been findings that CCHF infections usually occur in geographies with hot climate, where land farming is common. Many cases are reported from areas with increased animal farming where agricultural workers regularly come into contact with livestock [4]. It is likely that in endemic areas, infected livestock that notably remain asymptomatic may serve as virus reservoirs. In Kosovo, 32.6% while in Azerbaijan Shargi province of Iran, 25-80% of the sheep population was found seropositive during 1975–1999 survey for CCHF respectively [19]. This necessitates the importance of generating information on the prevailing tick vectors in our country and their disease association to CCHF infections. Similarly, most of the cases in Turkey during 2005 were either the result of tick-bite or patients who had viral exposure through livestock handling [20]. To date serological surveys of this nature have not been possible in Pakistan, although CCHF cases reported in Karachi city were linked to farming practices with sheep and goats brought from Baluchistan to Sindh province [21]. Similarly, large scale epidemiological surveys are required to study tick fauna in the context of CCHF infection. This will be important in defining hotspots of disease and constructing risk factors associated with particular regions. Such information will also help to define the range of tick species capable of hosting and transmitting CCHFV to humans, ultimately building evidence for vector and disease control programs that can directly support public health campaigns for populations who live in endemic areas.

Genetic sequencing of CCHFV genome provides knowledge of virus lineages which is a useful molecular epidemiological information and helps to understand risk areas and the transmission patterns of viruses [22], in addition to the long range relationships between outbreaks of disease. The genetic information of CCHF viruses reported from Pakistan is very limited due to lack of laboratory facilitates, however, the information available to date represents data of samples collected intermittently during 1976, 2000, 2001 and 2002 which were sent to international laboratories i.e. South Africa (National Institute for Communicable Diseases, Sandringham) and US-Centers for Disease Control and Prevention. Genetic characterization of all such viruses revealed that they are phylogeneticaly clustered with the viruses from neighboring Middle East Asian countries such as Iran, Afghanistan and UAE when their partial S-gene segments are compared [23]. Beyond the year 2002, no genetic information of CCHF viruses have been available despite the increased number of cases every year [16]. Our findings presented here further substantiate the existence of Asia-1 genotype in Pakistan explaining its vast endemicity in this region.

The recent report of a CCHF virus (strain; AFG09-2990) from Afghanistan showing 100% S-segment identity with the Pakistani strains [24] provides support for the close transmission links between these neighboring countries. It has been reported that Iranian strains also hold close similarity to Pak-Matin strain (Accession No. U75678) in Pakistan, indicating shared transmission sources between the two countries [17]. Likewise, another study analyzed the random interchange of CCHFV sequences between countries and found that Asia-1 genotype moves freely between Pakistan and Iran [14]. Tahmasebi *et al*. support such assumptions by finding that all CCHF viruses from Hamadan province of Iran clustered together with the CCHFV strains of Pakistan [17,19] proving evidence that the unchecked and uncontrolled animal movements between these countries remain a source of sustained and constant introductions of CCHFV to naive populations [25]. In the same way, animal trade between Pakistan and Middle Eastern countries also provide sources for the importation of CCHF virus from Pakistan to other countries, including UAE as evidenced by an outbreak in Dubai and was found linked to the transportation of cattle from Pakistan [26]. The Madagascar CCHFV strain isolated from *Boophilus microplus* species is believed to be introduced through cattle imported from Asia as these tick species occur primarily in Pakistan and India [23]. Two Chinese isolates (strain Fub900009 and C-68031) of CCHF virus have shown close homology with the viruses from Pakistan indicating frequent livestock trading or cultural and economic exchanges [27,28]. The presence of viruses with similar genetic make-up but in different geographical areas indicates the movement of CCHF virus-infected livestock or uninfected livestock carrying infected ticks due to considerable movement of sheep and goats from Pakistan into the Arabian Peninsula particularly at the time of religious festivals [7]. All such reports highlight that the CCHFV is not only contained within specific areas of Pakistan but has contributed to the transmission of virus across borders especially to the geographically neighboring countries [1]. Likewise, unlawful animal trade and uncontrolled population movements occur between Pakistan and Afghanistan through Quetta city due to the very similar cultural and tribal civilizations inherent across the bordering areas of both countries. Baluchistan province is a major source of animal’s skin and hides production, and also serves as a corridor for receiving skins and hides from Southern Afghanistan and Southeastern parts of Iranian Baluchistan via the Taftan gateway [29].

These findings provide significant evidence of frequent animal trade and movements and call for a dire need of strict quarantine regulations to be devised to ensure CCHFV screening of all animals to be transported from Pakistan to other world regions. More detailed tracing of animal movements, their role as potential disease carriers along with the analysis of virus genome sequences should be undertaken. This will provide beneficial outcomes due to the fact that assessment of livestock antibodies against CCHFV is one of the best indicators of CCHF risk to humans as well as effectively contribute to monitor the risk of CCHF in non-endemic regions [19]. Similarly, due to importance of migration patterns of CCHFV, more resources must be allocated for improved surveillance, sampling and collecting genetic information through sequencing of CCHFV strains [14].

Our study limitations include the genetic analysis based on partial S-gene sequence due to very low quantity of sample and insufficient resources, however, it can correctly define the genetic classification of CCHF viruses into seven distinct genotypes as utilized by previous studies [1,14,30,31].

## Conclusions

The migratory livestock population, carrying infection and/or the infected tick vector, serves as a major source of repeated introductions and continued transmission of CCHF to new flocks or areas. Maintenance of the virus in these populations demonstrates that CCHFV transmission is very efficient and it is likely that the CCHF case burden will expand with the increased trend of farming and animal trading. More resources should be allocated for surveillance and laboratory diagnosis of CCHF, especially in the endemic areas of Baluchistan, so that outbreaks if any, can be effectively contained. Regular infections and disease outbreaks have been reported from Baluchistan for many years and this area is a continuous risk which warrants control and preventative interventions. While infected animal movements during religious events are not the sole source of CCHF transmission outside and within Baluchistan, it is evident that the close contact between livestock and the animal holders, poor awareness of the disease management among the health care professionals, lack of laboratory diagnosis and inadequate medical practices at hospitals are also major contributing factors of CCHF endemicity and transmission within the country especially Baluchistan.

## Competing interests

All the co-authors declare that they have no competing interests.

## Authors’ contributions

SSZZ: Conceived and designed the study. AK, S Sharif, MSR: Performed the experimental work. MMA, S Shaukat, MA: Carried out the molecular genetic studies and phylogenetic analysis. SSZZ, MMA: Wrote the manuscript. All authors have read and approved the final manuscript.

## Pre-publication history

The pre-publication history for this paper can be accessed here:

http://www.biomedcentral.com/1471-2334/13/201/prepub
